# Study of silica-based intrinsically emitting nanoparticles produced by an excimer laser

**DOI:** 10.3762/bjnano.10.19

**Published:** 2019-01-16

**Authors:** Imène Reghioua, Mattia Fanetti, Sylvain Girard, Diego Di Francesca, Simonpietro Agnello, Layla Martin-Samos, Marco Cannas, Matjaz Valant, Melanie Raine, Marc Gaillardin, Nicolas Richard, Philippe Paillet, Aziz Boukenter, Youcef Ouerdane, Antonino Alessi

**Affiliations:** 1 Univ Lyon, UJM-Saint-Etienne, CNRS, Graduate School Optics Institute, Laboratoire Hubert Curien UMR 5516, F-42023, Saint-Etienne, France; 2 Materials Research Laboratory, University of Nova Gorica, Vipavska 11c 5270-Ajdovscina, Slovenija; 3 CERN, CH-1211 Geneva 23, Switzerland; 4 Dipartimento di Fisica e Chimica, Università di Palermo, I-90123 Palermo, Italy; 5 CEA, DAM, DIF, F-91297 Arpajon, France

**Keywords:** Ge-doped, laser ablation, nanomaterials, optical materials, silica

## Abstract

We report an experimental study demonstrating the feasibility to produce both pure and Ge-doped silica nanoparticles (size ranging from tens up to hundreds of nanometers) using nanosecond pulsed KrF laser ablation of bulk glass. In particular, pure silica nanoparticles were produced using a laser pulse energy of 400 mJ on pure silica, whereas Ge-doped nanoparticles were obtained using 33 and 165 mJ per pulse on germanosilicate glass. The difference in the required energy is attributed to the Ge doping, which modifies the optical properties of the silica by facilitating energy absorption processes such as multiphoton absorption or by introducing absorbing point defects. Defect generation in bulk pure silica before nanoparticle production starts is also suggested by our results. Regarding the Ge-doped samples, scanning electron microscopy (SEM) and cathodoluminescence (CL) investigations revealed a good correspondence between the morphology of the generated particles and their emission signal due to the germanium lone pair center (GLPC), regardless of the energy per pulse used for their production. This suggests a reasonable homogeneity of the emission features of the samples. Similarly, energy dispersive X-ray spectroscopy (EDX) data showed that the O, Ge and Si signals qualitatively correspond to the particle morphology, suggesting a generally uniform chemical composition of the Ge-doped samples. No significant CL signal could be detected in pure silica nanoparticles, evidencing the positive impact of Ge for the development of intrinsically emitting nanoparticles. Transmission electron microscope (TEM) data suggested that the Ge-doped silica nanoparticles are amorphous. SEM and TEM data evidenced that the produced nanoparticles tend to be slightly more spherical in shape for a higher energy per pulse. Scanning transmission electron microscope (STEM) data have shown that, regardless of size and applied energy per pulse, in each nanoparticle, some inhomogeneity is present in the form of brighter (i.e., more dense) features of a few nanometers.

## Introduction

In material science, laser–matter interaction encompasses not only the study of basic mechanisms but also material machining/engineering. Such emphasis is partially related to the increasing need for optical, photonic and medical devices used in telecommunication, medicine, sensing and imaging applications [1–2] as well as in biology, sustainable development, chemistry and physics. In most cases, micro- or nanostructures are nowadays required in all these fields, thus the study of their production and characterization has received considerable attention [1–4]. In the wide world of nanotechnology, silica-based nanosystems have attracted significant interest as proved by the various proposed applications in many domains [5–13]. The production of nanoparticles can be performed using both top-down and bottom-up procedures [12]. In the first case, the procedure starts from bulk materials to obtain nanoparticles, whereas in the second case, the procedure starts from atoms and often follows a chemical procedure to build up the nanomaterial. In this paper, we highlight some details on laser irradiation of bulk materials for the production of nanoparticles, where the material is removed from the target due to energy absorption from the laser. For this reason, the specific process of material removal depends on the optical characteristics of the target at the incident laser wavelength, on the laser pulse duration, intensity, repetition rate and other factors [4,14–17]. Energy absorption can take place through linear or super-linear processes [14], the first of which dominates in the case of an opaque target, when the laser intensity is low and/or the pulse duration is on the time scale of nanoseconds or longer. The second type of process is responsible for the absorption in transparent medium and is usually active for ultrashort laser pulses characterized by high intensity [14]. It is noteworthy that defects in or doping of a material such as silica can significantly change the optical absorption spectrum [15,18–19]. Indeed, this can result in additional absorption bands [19] and/or change the band gap [18]. For example, it was suggested that for long-duration laser pulses, point defects can provide “seed” electrons for avalanche ionization [14,20]. The feasibility of ablating pure silica has been demonstrated using lasers of different wavelengths and pulse durations ranging from tens of nanoseconds down to hundreds of femtoseconds [15]. It was also proposed that the damage is related to melting, boiling or fracture of the sample surface for laser pulses longer than 50 ps and to ablation for pulses with duration shorter than 10 ps [20]. In both cases, the production of nanoparticles was not investigated. In general, the ablation can take place in different ways such as explosion, evaporation, spallation or fragmentation [4]. Different from other studies [15,20], the purpose of the present investigation is to provide data concerning the production of pure and Ge-doped silica nanoparticles; thus it is worth highlighting some of the effects of the Ge doping of silica. Indeed, the Ge atoms are substitutional to Si in the glass network and the addition of Ge decreases the silica band gap [18,21–23]. The presence of Ge is associated with the appearance of new structures of optically active point defects such as the so-called germanium lone pair center (GLPC) [19,24]. This defect is responsible for an absorption band at ≈5.1 eV (≈240 nm) and for emission activity at ≈3.2 eV (≈390 nm) and ≈4.3 eV (290 nm) [19,24]. It is relevant to mention that the GLPCs are known to play a key role in the photosensitivity of the Ge-doped silica [25–26] used in fiber Bragg grating (FBG) manufacturing [27–28]. Moreover, in [29] the authors reported the multiphoton generation processes of point defects in silica, containing GLPCs, using laser excitation at wavelengths inside their absorption band, even for a low content of Ge and GLPC. We also emphasize that we have recently shown that nanoparticles can be generated by nanosecond pulsed KrF laser irradiation (140 mJ per pulse) from preform materials characterized by high Ge content and GLPCs [18]. In the present manuscript we report the possibility to generate nanoparticles from Ge-doped silica (produced by PCVD method [30]) using a lower energy per pulse, showing that for the used pure silica sample [31] a higher energy is needed. The acquired results confirm the positive role of the Ge doping, which facilitates the material removal by reducing the glass band gap and the presence of the Ge-related defects. By comparing the samples produced with different energies per pulse we provide evidence for the production of similar nanoparticles. Extending the previous studies we also show the amorphous nature of the nanoparticles produced by KrF irradiation.

## Results

In Table 1, we report the characteristics of the investigated samples. In particular, the sample name, the amount of Ge doping and the employed energy per pulse are indicated.

**Table 1:** Sample name, Ge content and employed energy per pulse.

Sample	Ge content (weight %)	Energy per pulse (mJ)

A	20	33
B	20	165
C	0	400

**Figure 1:**
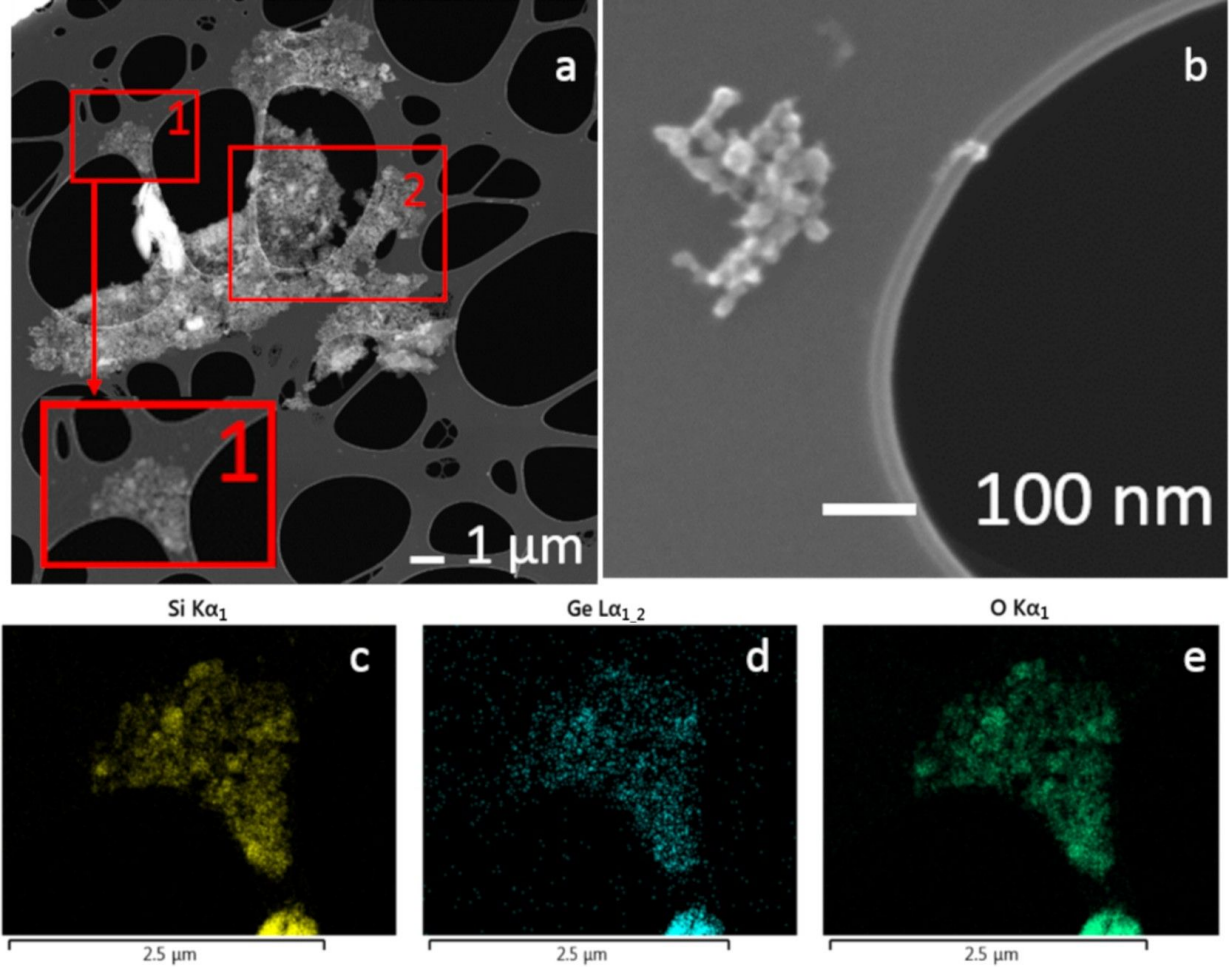
Sample A (SiO_2_+Ge, 33 mJ/pulse); a) SEM image of an agglomerate/aggregate of nanoparticles, inset zoom of the square 1; b) SEM image of a small group of nanoparticles; c), d) and e) EDX mapping of the Si Kα_1_, Ge Lα_1,2_ and O Kα_1_ lines, respectively, recorded in the zone indicated as square 1 in panel a.

Figure 1a illustrates a scanning electron microscopy (SEM) image of an agglomerate/aggregate of nanoparticles recorded for the sample A. From this image, its inset and panel b, we can observe the presence of nanoparticles of different sizes, from several tens up to hundreds of nanometers. Furthermore, some microparticles are also present. Similar results were obtained when surveying other parts of the same sample. In panel b a small group of nanoparticles of a few tens of nanometers is shown, which is a feature frequently observed in the sample. To deeply investigate our sample in Figure 1c,d and e we report the energy dispersive X-ray spectroscopy (EDX) signals of the Si Kα_1_, Ge Lα_1,2_ and O Kα_1_ lines, respectively. The data have been recorded for the group of nanoparticles inside the red square named 1 in panel a. These three images indicate that all the three elements are detectable. As expected, the signals of the three elements are higher when the quantity of the sample is larger in the morphological image (SEM), implying a higher amount of Ge, O and Si atoms. On the other hand, the chemical composition appears to be qualitatively homogenous for sample A.

**Figure 2:**
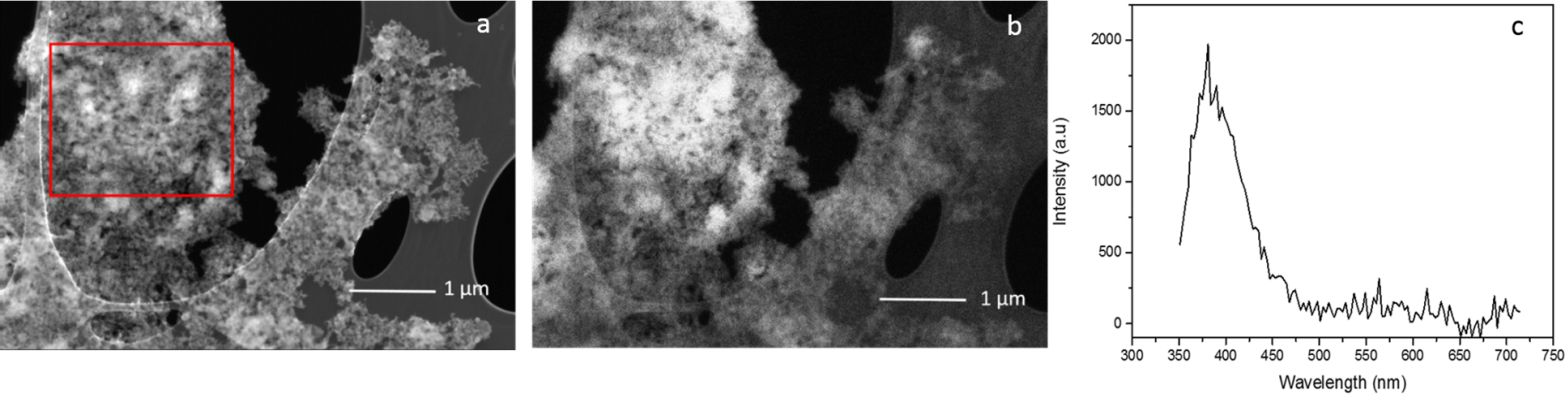
Sample A (SiO_2_+Ge, 33 mJ/pulse); a) SEM image of the zone indicated as square 2 in Figure 1a; b) CL panchromatic map of the same region; c) CL spectrum recorded in the zone marked by the red square in panel a.

In Figure 2a and 2b we compare, as an example, the SEM and the CL images recorded for the nanoparticle group in the region labelled 2 in Figure 1a. In particular, we observe a high correspondence between the morphological features of the nanoparticles and the CL panchromatic signal. Indeed, the entire set of particles emits a CL signal with a larger amplitude when a larger amount of particles is detected by the SEM. We underline that similar results were obtained in other parts of the same sample, and only small dissimilarities between the SEM and CL images are observed when the amount of nanoparticles is low. This effect can be attributed to the detection limit of the CL technique.

The origin of the CL signal has been investigated by spectroscopy. Figure 2c presents the CL spectrum recorded for the group of nanoparticles visible in the red square in panel 2a. The emission spectrum is clearly dominated by an emission band located around 400 nm, with spectral features corresponding to the those associated with the so-called GLPCs [18–19,24]. Such results agree with previously published works [18].

**Figure 3:**
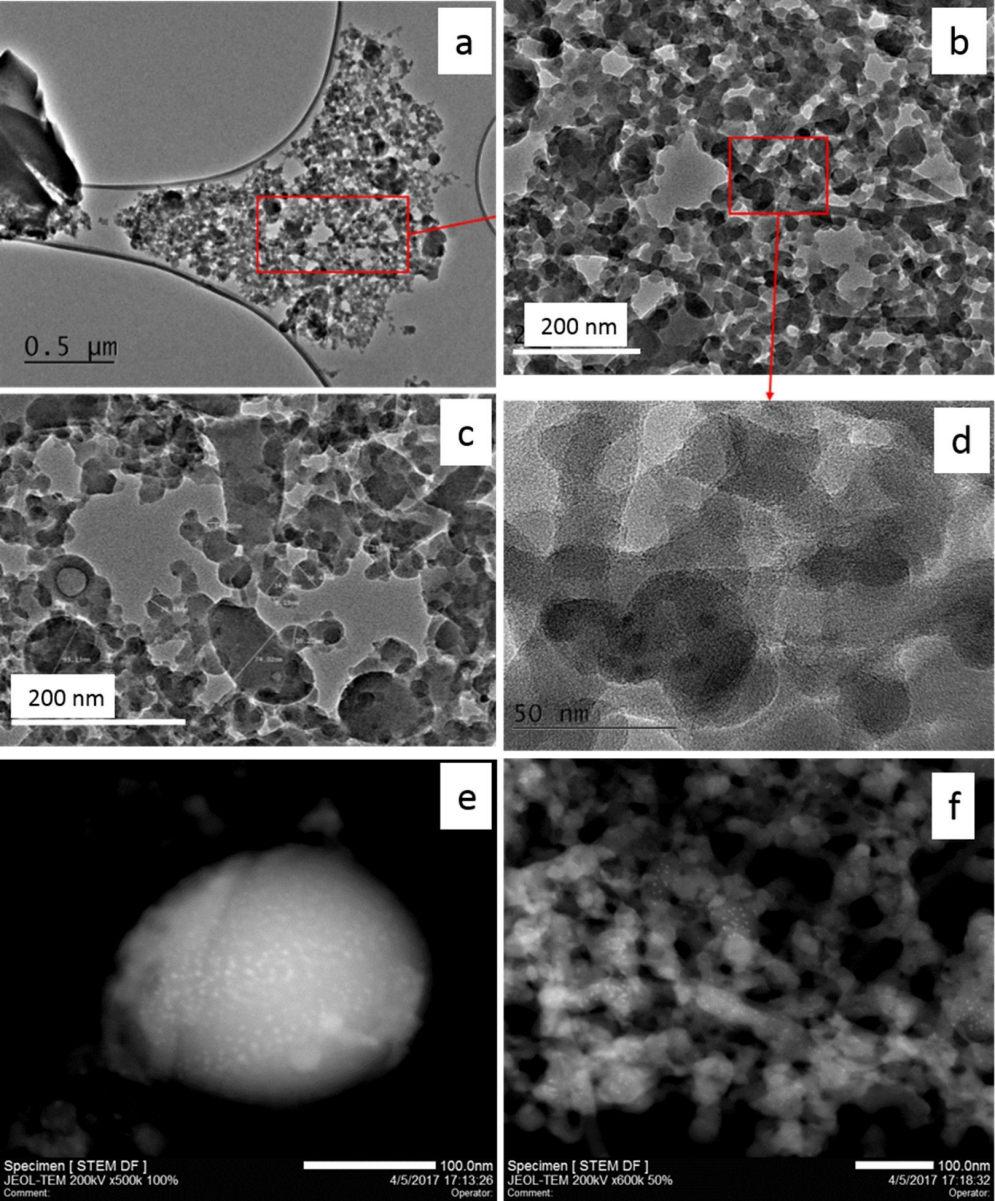
Sample A (SiO_2_+Ge, 33 mJ/pulse); a) TEM image of the region labeled 1 in Figure 1a; b) details of the red square of panel a; c) image of nanoparticles with different sizes; d) details of the red square of panel b; e) STEM image of a single, large nanoparticle; f) STEM image of an agglomerate/aggregate of small particles.

To further characterize the morphology of the produced nanoparticles, we recorded transmission electron microscopy (TEM) and scanning transmission electron microscope (STEM) images of the samples. Figure 3a illustrates the TEM image recorded for the same nanoparticles previously studied by EDX and SEM (red square named 1 in Figure 1a. Figure 3b displays the central part, evidencing the different shapes of the obtained particles and their states of aggregation/agglomeration. Another group of particles (Figure 3c) illustrates the variety of observed sizes and shapes. In this image, we can distinguish some spherical nanoparticles among other nanoparticles with irregular shapes. For both spherical and irregular particles, a broad size distribution is measured. Indeed, we have spherical particles with diameter of ≈10 nm up to particles of about 100 nm.

Similarly, for the irregular ones the sizes range from a few tens up to about 100 nm. The state of aggregation/agglomeration is also evidenced. More results are further highlighted in Figure 3d in which the particles seem to be merged or at least feature large contact regions. Furthermore, this image is recorded with a sufficiently high magnification to evidence the absence of a crystalline structure, indicating that the produced particles are amorphous. Figure 3e and 3f show the dark-field STEM images recorded for large nanoparticles and for a group of small ones detected in sample A. In both images we note the presence of several brilliant spots. Such spots are present independent from the particle size, and their dimensions are of a few nanometers. Moreover, in Figure 3e these features seem absent from the external edge of the nanoparticle, whereas they are more numerous in the internal part. We note that such nanoparticle features cannot be evidenced by SEM measurements.

**Figure 4:**
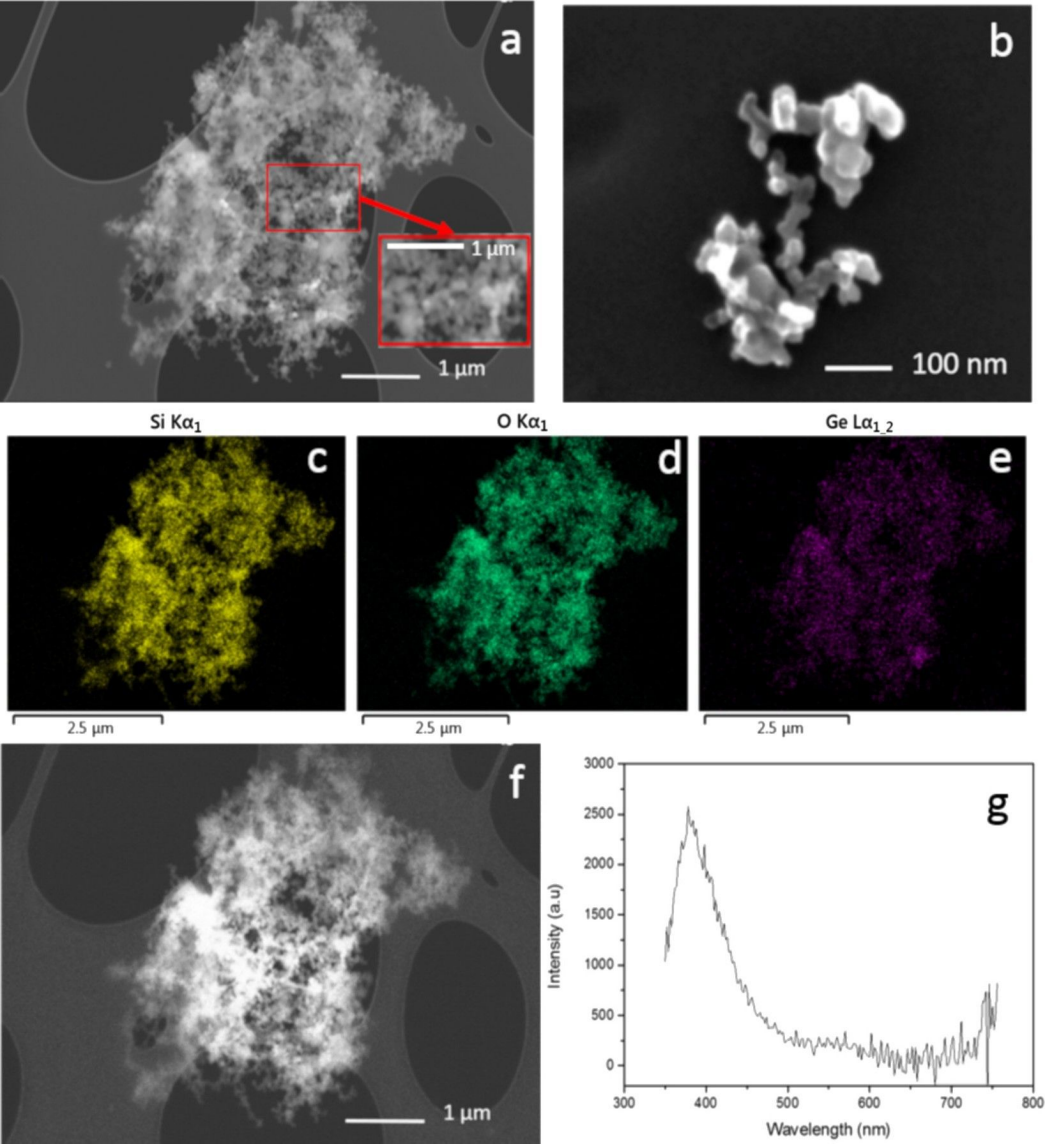
Sample B (SiO_2_+Ge, 165 mJ/pulse); a) SEM image of an aggregate/agglomerate of nanoparticles, inset zoom of the red square; b) SEM image of a small group of nanoparticles; c), d) and e) EDX mapping of the Si Kα_1_, O Kα_1_ and Ge Lα_1,2_ lines, respectively, acquired for the nanoparticles shown in panel a; f) CL panchromatic image of the same nanoparticles; g) CL spectrum of the area in the red outline in panel a.

Aiming to better understand the role of the energy laser pulse, we investigated a second sample produced with a higher energy per pulse, which is similar to the one used in [18]. SEM, EDX and CL data are shown in Figure 4. In detail, panel a, its inset, and panel b show the SEM data acquired for large and small groups of nanoparticles, respectively. Such images show the presence of agglomerates/aggregates of particles with size ranging from tens to hundreds of nanometers. As for sample A, we recorded the EDX image monitoring the Si, O and Ge related signals. An example of this investigation is illustrated in Figure 4c–e for the three elements, and the data are recorded for the same aggregate/agglomerate reported in Figure 4a. Once again, we observe a good correspondence between the morphology, studied by the SEM, and the EDX signals of the Si, Ge and O. Even in this case these elements appear quite uniform. This is additional information that was not possible to obtain in [18]. Figure 4f illustrates the CL mapping of the same group of nanoparticles.

The comparison with the SEM image (Figure 4a) provides evidence for a correspondence between the CL signal and the spatial distribution of the nanoparticles. Indeed, regions containing a larger amount of nanoparticles show higher CL signal. Similar results were obtained for the various investigated zones of sample B. For isolated nanoparticles or too small agglomerates, the CL signal is under the detection limit. The origin of the CL signal of Figure 4f has been determined by acquiring a CL spectrum in the zone marked by a red square in Figure 4a. Similar to sample A, and in agreement with previous data [18], the CL spectrum is dominated by an emission band peaked at about 400 nm having spectral features close to those of the GLPCs.

**Figure 5:**
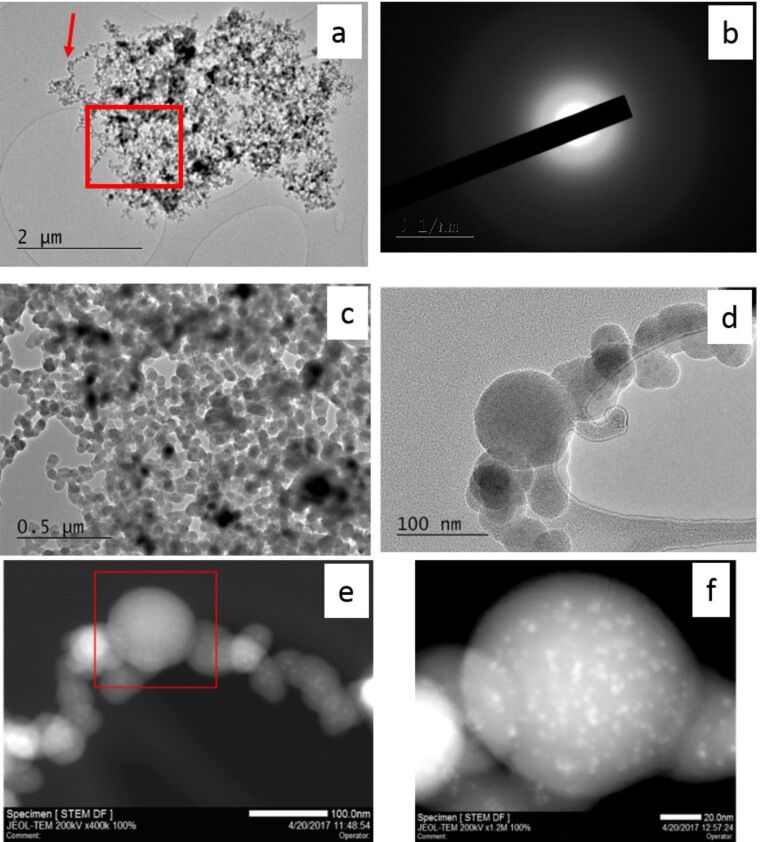
Sample B (SiO_2_+Ge, 165 mJ/pulse); a) TEM image of the same agglomerate/aggregate of nanoparticles of Figure 4a; b) diffraction pattern recorded for these nanoparticles; c) details of the zone inside the red square in panel a; d) details of the zone indicated by the red arrow in panel a; e) STEM image of the nanoparticles of panel d; f) STEM image of the largest nanoparticles of observed in the red square of panel e.

In Figure 5a, we report a TEM image of some nanoparticles previously investigated by SEM/EDX/CL. Figure 5b illustrates the diffraction pattern generated by the nanoparticles of this sample. The absence of any diffraction spot confirms the amorphous state of the nanoparticles. In Figure 5c and 5d we report two images recorded with increasing the magnification. Panel c shows the nanoparticles that belong to the zone marked by the red square of panel a, whereas panel d shows the few nanoparticles indicated by the arrow in the panel a. The TEM image of Figure 5c shows the presence of a large amount of spherical nanoparticles having an average diameter of about 50 nm, even if larger particles with diameter above 100 nm are also detected. An example is reported in Figure 5d that illustrates the presence of large and small particles. Such an image also confirms the state of aggregation/agglomeration of the investigated system. Figure 5e reports the STEM image of the same group of nanoparticles displayed in panel d. In this image, we note again the presence of bright spots detected in the different size particles. Increasing the magnification we recorded a STEM image (reported in Figure 5f) of the biggest nanoparticle, evidenced by a red square in panel e. The acquired image is reported in Figure 5f. From this image we note that the spot sizes are of a few nanometers (2–4 nm). We observe again that in the external part of the particle, such spots appear to be absent, whereas they are clearly observed in the internal part, suggesting that they could be mainly located inside the nanoparticles rather than on their surface.

Some microparticles were also detected in sample B. Similarly, although in the SEM and TEM images, the spherical particles appear more frequent than in sample A, irregular particles of different sizes were detected, too.

**Figure 6:**
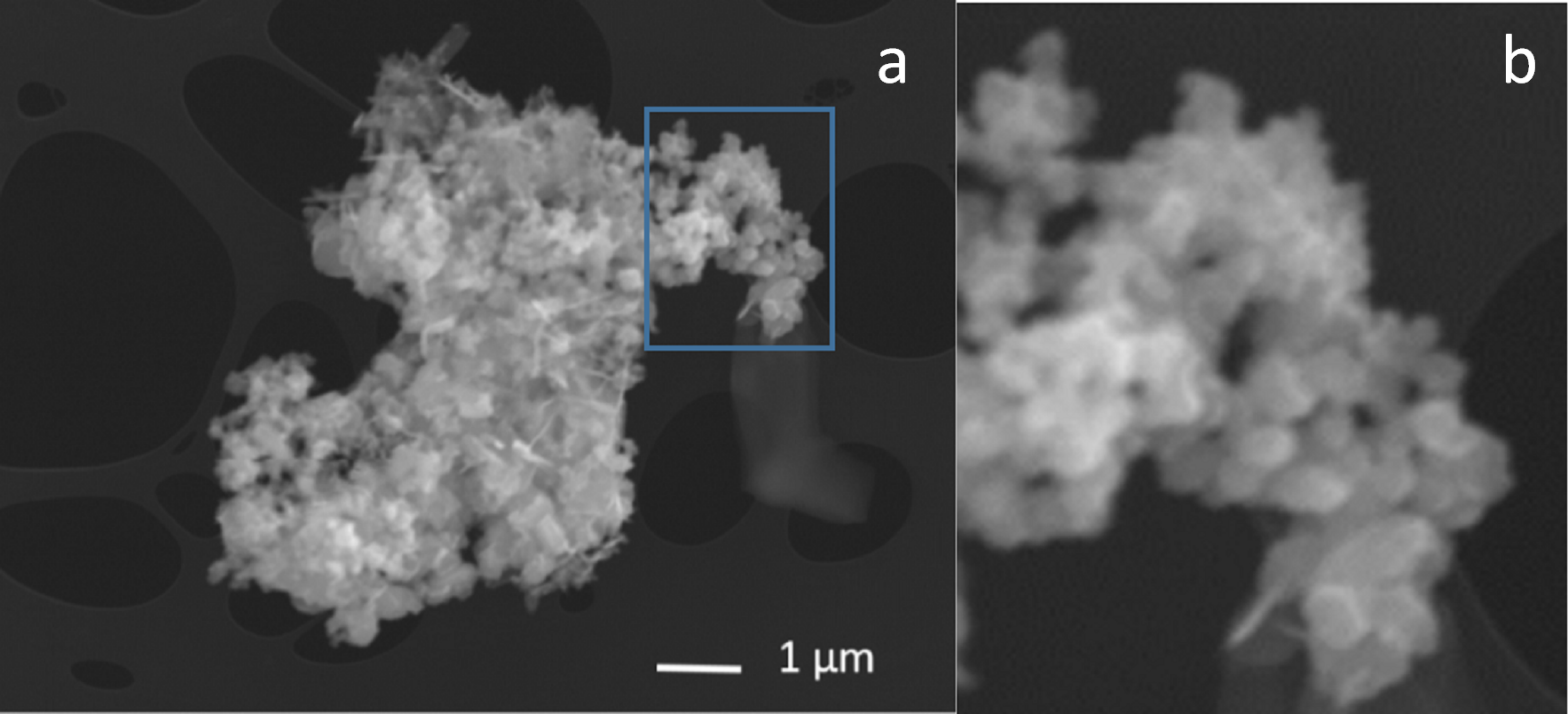
SEM image of an aggregate/agglomerate of sample C (SiO_2_ 400 mJ/pulse); b) details from panel a.

The last sample that we studied is a pure silica material, as reported above, where the employed energy per pulse was about 400 mJ. One of the SEM images recorded for this sample is reported in Figure 6a, which illustrates a large agglomerate/aggregate of particles and some flakes. In panel b we show details of a part (framed in blue in panel a) of such image. Such a detailed image allows us to illustrate the presence of nanoparticles of hundreds of nanometers in diameter and some with a diameter of under 100 nm. The EDX signals of such a sample revealed the presence of iron. This contamination, probably due to damage of the sample holder with the beam external tail, limited the TEM characterization of sample C. As a consequence the presence of the produced nanoparticles (one of the main aims) is verified by the SEM image. Secondly, it was also possible to investigate the presence of the CL signal and to perform the comparison with that of the Ge-doped nanoparticles. In particular, the CL spectra did not show the presence of any clear luminescent features above the noise background.

## Discussion

In the present investigation provide evidence for three main results: i) the comparison of samples produced by only varying the starting energy per pulse showed the generation of nanoparticles in Ge-doped silica at a laser energy per pulse lower than that in previous investigations [18]; however, we can not exclude that lower values of fluence (adding filters to attenuate the starting laser beam, changing the spot size or other irradiation parameters) can induce generation as well; ii) the possibility to generate nanoparticles from undoped silica, using higher energy per pulse; and iii) improved structural and morphological characterization of the nanoparticles using TEM and STEM, as well as detecting the Ge atoms with EDX measurements. The suggestion that the addition of Ge atoms (which results in lowering the band-gap and introducing defect species with an absorption band at 5.1 eV) is able to facilitate the nanoparticle generation is confirmed by the high difference (about one order of magnitude) in the energy per pulse needed to obtain an observable material removal in the case of the pure silica (sample C) – at the same energy we observed no material removal in pure silica for the Ge-doped samples.

To further understand the generation of the nanoparticles, it is relevant to note that for the Ge-doped samples only a few laser pulses (≈1 s of irradiation) were needed even at the lowest energy (33 mJ, sample A), to observe (by the naked eye) material removal; in the case of the pure silica samples, more pulses (10–20 s of irradiation) were needed and the irradiated parts become very white before the removal of matter. Such phenomenon, if present, was too fast to be observed in the Ge-doped samples. This phenomenon could indicate that a large amount of absorbing defects have to first be generated to achieve a sufficient absorption of energy at the wavelength of the KrF laser. Such an effect can be compared to the incubation effect previously reported in [17]. This has a lower impact or takes place faster in Ge-doped glasses as a consequence of the band gap shift to lower energy and the presence of GLPC (see [18] for the optical absorption spectrum of the employed Ge-doped samples).

Actually, the decrease of the band gap implies that the order of the multiphoton absorption can be lowered and the presence of defects with optical transition at ≈5 eV allows a standard absorption from the excitation laser, eventually followed by a second absorption from the real excited state of the defects (the first excited state of GLPC, responsible for the 5 eV band, has a lifetime of about 4 ns at room temperature [23–24]) to the conduction band (ionization of GLPC). It should also be noted that irradiation of Ge-doped silica, in general, and with a KrF laser, as in the present case, induces the generation of other defects and increase of the absorption coefficient in the UV range [23,25,28].

The higher efficiency of energy transfer to the network in Ge-doped silica is also suggested by the data of [29] and by the known sensitivity of this kind of silica to UV irradiation even from the point of view of the matrix reorganization [28]. As suggested in [18] and considering the studies reported in [14], the long time duration of our laser pulse suggests that the energy was transferred to a sample volume larger than that where the laser was focused. Furthermore, the presence of microparticles can be justified by the fact that in some cases, the sample featured some explosion. Regardless, the comparison between the pure and the Ge-doped samples highlight the advantage of having Ge atoms in the material thanks to their ability in forming oxygen-deficient centers such as the GLPCs [19,32–33]. Moreover, we note that independent from the energy per pulse, in the two Ge-doped samples, the CL is due to the GLPC without additional contributions from other Ge or intrinsic related emitting defects. As a consequence, the contribution of this defect type appears fundamental for the production of emitting nanoparticles. Since in pure silica we did not detect a CL signal and since an increase in the laser power by a factor of about 5 in the Ge-doped silica nanoparticles did not evidence a detectable, additional component in the CL spectra, it is concluded that enhancing the contributions from the other defects seems difficult by simply changing the laser energy employed during the production.

Considering the two Ge-doped samples, there is only a small tendency in sample B to form more regular (spherical) particles. In this case, explosion of the sample occurred more frequently during irradiation. This experimental result indicates that it would be difficult to obtain monodisperse, regularly shape nanoparticles by increasing or decreasing the energy per pulse of a nanosecond pulsed laser with a spot size of millimeters.

In the following we consider that the particles of irregular shape are generated mainly during explosion events and that spherical particles are induced by the melted and then rapidly quenched events. On one hand, increasing the energy per pulse allows a larger part of the sample to absorb a sufficient amount of energy to be melted. On the other hand, this growth of the melted mass and possible increase of the induced thermal gradient, which together can create stress or inhomogeneity and can induce explosion, implies a higher tendency to explosion.

Furthermore, the laser spot has a spatial profile (typically with a maximum in the center) which implies that different regions, within the spot, are irradiated differently. Our data indicate that for low energy per pulse (or fluence) the particle shape tends to be irregular. The use of the same mask could limit the contribution of the irradiated part far from the center (thus with low local fluence), which seems to contribute to the generation of irregular particles.

In any case, the presence of the Ge atoms and the consequent increase of the sensitivity to laser irradiation could be combined with other ablation procedures using a UV ns pulsed laser as the one reported in [34]. In regard to previous studies [18], we improved the EDX analysis providing evidence of the essential correspondence between the morphology and the spatial distribution of the Ge, Si and O content independent from the energy per pulse. Such results suggest that the produced nanoparticles should be constituted of Ge/Si dioxide. The fact that we detected a GLPC emission with spectral features very close to those detected in Ge-doped silica supports this possibility in the sense that Ge-doped silica regions do exist and from the CL signal they are uniformly distributed. The presence of Si, Ge or Ge/Si richer regions can not be totally ruled out, higher spatial resolution is required for further investigation.

Our TEM experiments also highlighted the amorphous nature of the obtained nanoparticles for samples produced with various values of energy per pulse. Such results indicate that crystallization is not induced, so even if high energy absorption can induce melting of part of the sample, the quenching is sufficiently fast to avoid the formation of large crystalline regions.

The obtained nanoparticles differ in size and are in general larger than a few tens of nanometers. Such dimensions do not represent a relevant issue for some applications, as discussed in [5–6,13,35–37], and the irregular shape might not be an insurmountable problem from an applications point of view [37]. Independent from the employed energy per pulse and from the nanoparticle size, the STEM images evidenced the presence of some bright spots. In dark-field STEM mode this implies that the bright spots indicate a higher density or higher concentration of high-Z elements (or both). Such spots appear to be located in the internal part of the particle rather than at the surface. Their nature remains unknown at the moment, but a realistic hypothesis is that they are Ge-rich features such as Ge or GeO_2_ rich zones or densified parts for example. The presence of Ge-rich zones, even if of small size, has been proposed in Ge-doped bulk silica as a consequence of an imperfect mixing or composition fluctuations [22–23].

## Conclusion

In this work, we employed various techniques to produce Ge-doped and pure silica nanoparticles using nanosecond pulsed KrF laser irradiation. Our experiments demonstrated the possibility to generate nanoparticles using a laser pulse energy of about 33 mJ and 400 mJ for doped and pure silica materials, respectively. The difference in the required energy per pulse has been attributed to the high Ge doping level, which affects the optical properties of the silica. As a consequence of the presence of the GLPC, the emission activity was studied using cathodoluminescence measurements only for the Ge-doped nanoparticles. In addition to experiments using 33 mJ irradiation, an experiment was performed on Ge-doped glass with an energy per pulse of about 165 mJ. Comparing the samples produced with 33 mJ and 165 mJ (based on TEM images) we noted a slight tendency to form more spherical nanoparticles in the second case. In both cases we also noted a significant correspondence between the morphology studied by SEM images and those studied by cathodoluminescence, suggesting a certain homogeneity in the optical properties of both samples. The amorphous structure (as investigated by TEM) and qualitative homogeneity in chemical composition (as studied by EDX) of Ge/Si dioxide appears to be independent of the energy per pulse applied. Nevertheless, STEM data indicate the presence of some inhomogeneity inside each nanoparticle, likely arising from aggregation of elements or densification. Such a result is observed independent of the size or the energy used for their production. Thus, apart from the size, the produced samples are a set of nanoparticles with reasonable homogeneity in structure, chemical composition and emission features.

## Experimental

For the present investigation, we used two different types of silica bulk materials. The first one is doped with 20 wt % Ge, produced by plasma-activated chemical vapor deposition (PCVD) [23,30]. The second one is a pure silica synthetic commercial sample containing high OH content [31], similar to the one irradiated with a 500 fs 248 nm laser in [15]. The nanoparticle synthesis was carried out using a laser ablation (or irradiation) technique in liquid, which was successfully employed in the past on different materials [38–40].

In detail, employing a preparation procedure similar to that used in one of our previous studies [18], the samples were immersed in distilled water (the height of water above the sample was ≈1.5 cm) and exposed to the focused (a lens having a 20 cm focal length was employed) beam (diameter ≈1 mm, the effect of water on the focal length variation [40] and spot size at different distances from the focal point was considered) of a KrF pulsed laser (λ = 248 nm, pulse duration 30 ns, repetition rate 10 Hz, COMPex 110 from Lambda Physik). Two samples were prepared by irradiating the Ge glass at ≈33 and ≈165 mJ per pulse, and the obtained nanoparticle samples were labelled as sample A and B, respectively. The pure silica sample was exposed to ≈400 mJ per pulse and the obtained sample was labelled as sample C. Exposing the sample to ≈200 mJ we observed no material removal. In all cases we used a sample holder to keep the sample immersed in water far from the bottom of the container. All the samples were prepared by the same procedure by changing the energy per pulse of the laser, so that the ratio between the different evaluated fluences are not affected by errors of the spot size estimation. The maximum fluence is about 50 J/cm^2^ considering a uniform distribution on the spot surface and neglecting losses along the optical path in air and water. This latter assumption on transparency of the employed liquid is generally confirmed, even if multiphoton absorption by liquid or other effects are present [40].

The samples were moved along the plane orthogonal to the laser beam in order to refresh the irradiated region, and the laser focus was adjusted to keep it at the sample surface. A few seconds of irradiation were needed to observe the starting of the material removal from the silica sample.

SEM images were acquired with a JSM 7100F (JEOL) instrument, using an electron energy of 20 keV and probe current of ≈10 nA. The samples were not coated with a conductive layer as in [18]. The same instrument was used to perform EDX experiments using an X-Max 80 (OXFORD) detector, and cathodoluminescence (CL) spectroscopy and imaging was performed using a MONO-CL4 (GATAN) detector working in the spectral range 300–750 nm (1.7–4 eV). EDX, CL images and spectra were recorded using a current of ≈10 nA and an electron energy of 20 keV. Using these experimental conditions and in the investigated spectral domain, we did not detect any CL signal originating from the substrate.

TEM and STEM images were recorded with a JEM-2100F UHR (JEOL) device at 200 keV beam energy. STEM images were acquired with a dark-field annular detector.

For all the experiments we used holey-carbon-coated grids on 200 mesh copper from SPI, since these grids allow the characterization of the same zones along the samples with the different techniques. The thin network-shaped carbon film helps to recognize the location of the investigated particles. The suspensions resulting after the laser irradiation (distilled water + silica particles) were kept at about 90 °C to reduce the amount of water. Then, we shook the solution and immersed one grid in it, and left it on a heating plate that was preheated to 60 °C. The grids were supported by an aluminium stub for SEM/EDX/CL characterization.
